# Non-pharmacological interventions for reducing anxiety and depression symptoms among adolescents and young adults: Protocol for a scoping review

**DOI:** 10.1371/journal.pone.0351315

**Published:** 2026-06-23

**Authors:** Gerald Agyapong-Opoku, Belinda Agyapong, Temitayo Sodunke, Ejemai Eboreime, Andrew Greenshaw

**Affiliations:** 1 Department of Psychiatry, University of Alberta, Edmonton, Canada; 2 Department of Psychiatry, Dalhousie University, Halifax, Canada; 3 School of Health and Human Performance, Faculty of Health, Dalhousie University, Halifax, Canada; Keimyung University, KOREA, REPUBLIC OF

## Abstract

**Background:**

Anxiety and depression are leading causes of mental health-related disability among adolescents and young adults worldwide. These conditions often begin in adolescence and can persist into adulthood, with long-term consequences for individuals’ well-being, education, and social functioning. Although a growing number of interventions have been developed to address these concerns, they vary widely in format, setting, and target population.

**Objective:**

This scoping review aims to systematically map the range and characteristics of interventions targeting anxiety and depression among adolescents and young adults aged 10–24.

**Methods:**

This scoping review will be conducted following the Joanna Briggs Institute (JBI) methodological guidance for scoping reviews and reported in accordance with the PRISMA-ScR checklist. A comprehensive search will be undertaken across several electronic databases (PubMed, PsycINFO, Scopus, CINAHL, Web of Science, and Embase). Eligible studies will include any empirical research focused on interventions aimed at reducing symptoms of anxiety or depression among individuals aged 10–24 years. Two independent reviewers will select and chart the studies, and findings will be summarized using descriptive and thematic analysis.

**Results:**

The findings will provide an overview of interventions adopted to reduce anxiety and depression among adolescence across diverse settings, identify the most popular or commonly accepted interventions among this cohort and its modalities. The findings will also highlight the need for stakeholders and policymakers to support the design, implementation, and evaluation of targeted mental health strategies for adolescents and young adults.

**Dissemination:**

Findings will be published in a peer-reviewed journal and shared with stakeholders including researchers, clinicians, and policymakers. The results will inform future research, intervention development, and policy planning aimed at supporting the mental health of adolescents and young adults.

## Introduction

Globally, anxiety and depression pose significant challenges to adolescents and young adults, affecting, their academic performance, social relationships and overall wellbeing [1]. Anxiety and depression are usually comorbid and among the most common mental health disorders experienced by adolescents and young adults, with onset often occurring during this critical developmental stage [2]. These disorders are associated with substantial personal, academic, and societal burdens, including increased risk for substance use, poor educational attainment, unemployment, and suicide [3].

The global burden of mental health challenges in youth has been further exacerbated by recent stressors, such as the COVID-19 pandemic, which has intensified symptoms of anxiety and depression in this population [4,5], thus highlighting the urgent need for effective interventions. A recent scoping review reported increased prevalence rates of anxiety and depression [6]. In response to these rising trends, young people often engage in self-medicating behaviors, such as using tobacco or combining it with marijuana, in an attempt to alleviate their symptoms [7]. However, these coping mechanisms are maladaptive, carry significant health risks, and fail to address the underlying psychological conditions.

Pharmacological intervention (medications) has been the primary intervention utilized for depression and anxiety symptoms. Non-pharmacological interventions in relation to this scoping review will be defined as structured therapeutic strategies that aim to improve health outcomes without the use of medications (prescription, over-the-counter, or investigational drugs) as part of the intervention. Non-pharmacological interventions may include psychological or behavioural multidimensional rehabilitation, online approaches, physical, social, educational, digital, or lifestyle-based approaches that function independently of pharmacological treatment [8]. Evidence suggests that self-disclosure strategies may be effective for reducing depressive symptoms among adolescents and young adults (AYAs), particularly for those already diagnosed with depression, although no similar effects have been observed for anxiety [9]. Treatment of depressive disorders is among the most effective ways to reduce disease burden, with model estimations suggesting that wide-scale implementation of therapy, and collaborative care could decrease the burden by 10–30% [10]. However, access to traditional mental health care remains limited, especially for youth, due to structural barriers such as financial constraints, limited availability, stigma, and preferences for self-management [11].

Digital mental health interventions may offer promising alternatives to conventional care, particularly for AYAs facing such barriers, as they are well adapted to the use of mobile technology, especially text and email messages, which present a unique opportunity for an innovative way to offer support for their mental health [1]. For instance, digital support, including text messages, can be adopted to support the mental health of young adults and adolescents [12]. These practical and scalable interventions can support user autonomy while expanding access to evidence-based treatments [13]. Studies have demonstrated that internet-based interventions are not only feasible but also improve mental health outcomes [14]. Among these, web-based interventions grounded in cognitive behavioral therapy (CBT) and interpersonal approaches have shown effectiveness in preventing or reducing symptoms of depression and anxiety in young people [15–17]. Additionally, physical activity has been found to improve mood and cognitive functioning, while community-based interventions show promise in addressing mental health at a broader level [18].

Given the wide range of interventions targeting anxiety and depression in adolescents and young adults (AYAs), and the variability in their design, implementation, and outcomes, a comprehensive scoping review is warranted. Additionally, many reviews combine children, adolescents, and adults without clearly isolating findings for the 10–24 age group. There is a gap in evidence specifically synthesizing outcomes for adolescents and young adults as a distinct developmental population. This scoping review will provide a systematic overview of existing strategies, highlight patterns and gaps in the literature, and offer valuable insights to guide the development of more effective, evidence-based approaches for future research, policy, and clinical practice.

### Justification

While numerous interventions have been developed and implemented to address anxiety and depression in adolescents and young adults, including psychological therapies, digital tools, physical exercise, school-based programs, and community-led initiatives, the evidence base remains dispersed and inconsistent [19]. There is a limited comprehensive synthesis targeting the 10–24 age range, which has mapped the full range of interventions across settings and delivery modes for this specific age group.

A scoping review is therefore warranted to systematically explore and characterize the breadth of existing interventions for anxiety and depression among adolescents and young adults aged 10–24. This review will help clarify the types of interventions available, their contexts of delivery, populations targeted, and outcomes measured. It will also identify gaps in the literature and inform future research priorities, clinical decision-making, and policy development.

## Methods

This scoping review will be conducted using the methodological framework proposed by Arksey and O’Malley, which includes five key stages: identifying the research question, identifying relevant studies, study selection, charting the data, and collating, summarizing, and reporting the results [20]. The review will be guided by enhancements suggested by Levac et al. [21], particularly in refining the research question and ensuring methodological rigor as well as methodological guidance from the Joanna Briggs Institute [22,23]. There is conceptual overlap among the three frameworks. Generally, Arksey & O’Malley provide the foundational scoping review structure, Levac et al. enhance methodological rigor, and the JBI framework offers detailed guidance for data extraction and reporting.

### Identifying the research question

We aim to answer the following questions:

1. What are the non-pharmacological interventions for reducing anxiety and depression among adolescents and young adults aged 10–24?
2. What recommendations have been made in the literature for improving access to these interventions?

### Identifying relevant studies

A comprehensive and systematic literature search will be conducted to identify studies examining interventions for anxiety and depression among adolescents and young adults aged 10–24. The search will include peer-reviewed primary studies, guided by the Population–Concept–Context (PCC) framework recommended for scoping reviews [24,25]. Electronic databases to be searched are PubMed, PsycINFO, CINAHL, Scopus, Web of Science, and Embase.

The search strategy will use a combination of controlled vocabulary (e.g., MeSH terms) and free-text keywords related to the population (e.g., adolescents, “young adults”, youth), condition (e.g., anxiety, depression), and interventions (e.g., treatment, therapy, intervention, “psychological support”). An example search string for PubMed includes: (adolescent[MeSH Terms] OR adolescent* OR teen* OR youth OR “young adult*” OR “young age”) AND (anxiety[MeSH Terms] OR depression[MeSH Terms] OR “anxiety disorder*” OR “depressive disorder* OR “mental health”) AND (intervention* OR treatment* OR therapy* OR counseling OR “psychological therapy” OR “school-based intervention*). The search strategy will be adapted to suit each database’s indexing terms and interface requirements.

Restrictions, in the last ten years, will be applied to the publication date to capture the most recent intervention. Additionally, only studies available in English will be included in the final analysis. Furthermore, other studies will be identified by screening the reference lists of included articles and relevant reviews. The complete search strategies for each database will be documented in an appendix to ensure transparency and reproducibility.

### Study selection

#### Eligibility Criteria.

The inclusion and exclusion criteria for this scoping review are defined according to the PCC framework, consistent with the methodological guidance from the Joanna Briggs Institute [22,23].

### Population

This review will include studies involving adolescents and young adults, broadly defined as individuals aged 10–24 years, in line with the age range recognized by the World Health Organization [26].

### Concept

The review will focus on interventions aimed at preventing, reducing, or mitigating symptoms of anxiety and/or depression. Eligible interventions may include psychological therapies (e.g., cognitive-behavioral therapy, interpersonal therapy), digital health tools (e.g., mobile apps, online programs), school-based initiatives, peer support models, and community-based programs [3,27]. Interventions must have a clearly stated focus on mental health and explicitly target anxiety, depression, or both.

### Context

There will be no restrictions on geographic location, healthcare system, or intervention setting. Studies conducted in clinical, school, community, or digital/online environments will be included to reflect the diversity of service delivery models for this population [28].

### Types of sources

This review will consider primary research studies employing qualitative and quantitative methods including randomized controlled trials, quasi-experimental studies, cohort studies, and program evaluations. Systematic reviews and meta-analyses will be excluded as primary evidence but may be used to identify additional relevant primary studies.

### Exclusion criteria

Studies will be excluded if they [1] do not focus on anxiety or depression [2] involve populations entirely outside the 10–24 age range [3] include mixed interventions where medication is involved. However, studies involving mixed interventions will only be included if the non-pharmacological component is clearly isolated and analyzed separately [4] do not describe or evaluate an intervention or focus on pharmacological interventions; or [5] are editorials, opinion pieces, grey literature, or theoretical papers. Due to resource limitations, studies not available in English will also be excluded.

After completing the search for each database, the resulting articles obtained will be uploaded onto Covidence [29], a web-based software platform designed to aid systematic reviews. Covidence is designed to automatically remove duplicates as well as document all stages of the selection process. After initial screening of titles and abstracts, full texts of potentially relevant articles will be reviewed for final inclusion. The title, abstract, and full-text screening will be conducted independently by two reviewers, and discrepancies will be resolved through discussion or consultation with a third reviewer.

### Data charting

Data will be extracted using a standardized data charting form developed by the research team. Two reviewers will independently extract and categorize data from the included studies. Themes will be developed iteratively through regular discussion and consensus, with discrepancies resolved through reviewer deliberation. Inter-reviewer agreement will be assessed periodically to ensure consistency and rigor in the analytical process. Extracted information will include author(s), year of publication, study location, sample size, participant characteristics, intervention type and duration, mode of delivery, and reported outcomes as shown in Table 2.

### Collating, summarizing, and reporting the results

The relevant data will be organized into tables and validated by at least two members of the research team. The characteristics and results reported in each included article will then be summarized. The charted data will be analysed thematically to identify key trends and patterns across the literature, and agreed upon and standardized by the team. Interventions will be categorized by type (e.g., psychological therapy, digital tools, school-based programs), delivery method (e.g., individual vs. group, in-person vs. digital), and primary outcomes (e.g., symptom reduction, improved well-being). This structured synthesis will facilitate the identification of both well-established approaches and emerging trends in the reduction, mitigating, and prevention of anxiety and depression symptoms among AYAs. Multi-component interventions will be clarified by applying explicit classification criteria during data charting and synthesis. Interventions will be primarily categorized based on their dominant component, defined as the element most central to the intervention’s objective or most emphasized in the study description; however, where multiple components contributed substantively, interventions will also be secondarily categorized. This will ensure consistency; while allowing overlap to be transparently reported in the results, Table 1 outlines the planned timelines and milestones for the scoping review.

**Table 1:** Planned timeline and milestones for the scoping review.

Phase	Review Activity	Key Tasks	Expected Output	Timeline
Phase 1	Protocol Development	Finalize research objectives, eligibility criteria, and methodological framework based on Arksey & O’Malley and PRISMA-ScR guidance	Completed scoping review protocol	February – March 2026
Phase 2	Search Strategy Development	Develop and refine database search strategies with librarian support; pilot search terms	Finalized search strategy	April 2026
Phase 3	Literature Search	Conduct systematic searches across selected databases and export results to reference management software	Database of retrieved records	April 2026 – June 2026
Phase 4	Title and Abstract Screening	Independent screening of titles and abstracts by two reviewers based on inclusion and exclusion criteria	Preliminary list of eligible studies	July 2026
Phase 5	Full-Text Screening	Retrieval and detailed assessment of full-text articles; documentation of reasons for exclusion	Final list of included studies	August 2026
Phase 6	Data Charting	Extract and organize relevant study information using a standardized data extraction form	Completed data extraction dataset	September – October 2026
Phase 7	Data Synthesis	Conduct descriptive and thematic analysis of intervention characteristics, outcomes, and research trends	Tables, charts, and thematic summaries	November 2026
Phase 8	Reporting and Dissemination	Prepare and submit the final manuscript following PRISMA-ScR reporting standards	Submitted scoping review manuscript	December 2026 – January 2027

## Results

The results of this scoping review will provide a comprehensive mapping of the literature on non-pharmacological interventions aimed at reducing anxiety and depression symptoms among adolescents and young adults (10–24 years). Findings will be reported in accordance with the PRISMA-ScR guidelines to ensure transparency and methodological rigor.

**Fig 1:**
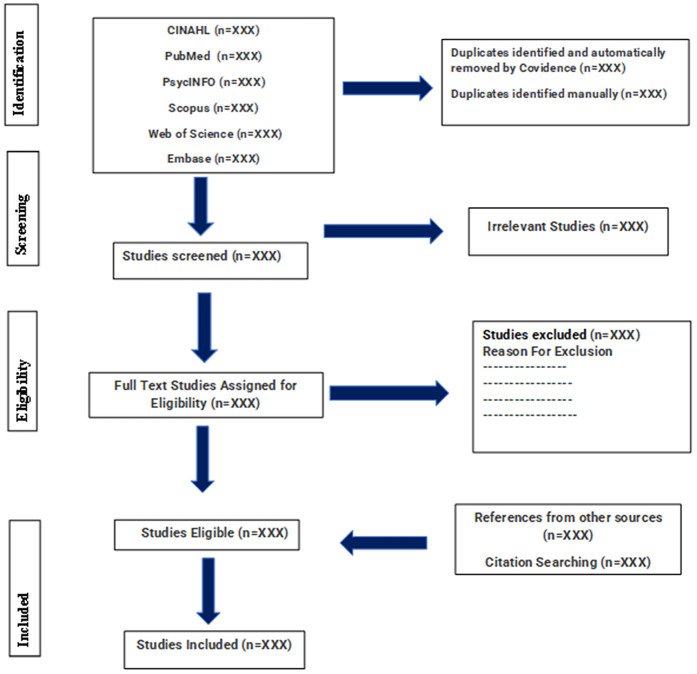
PRISMA Flow Diagram. CINAHL Plus: Cumulative Index of Nursing and Allied Health Literature. n: Number.

The study selection process will be presented using a PRISMA flow diagram outlining the number of records identified, duplicates removed, titles and abstracts screened, full texts assessed for eligibility, and studies included in the final review as shown in Fig 1 (if accepted, production will need this reference to link the reader to the figure). This will be accompanied by a narrative summary of the screening process and primary reasons for exclusion at the full-text stage. A descriptive synthesis of included studies will summarize publication trends over the past decade, study designs, geographic distribution, sample sizes, participant characteristics, targeted conditions (anxiety and/or depression), intervention types, and reported outcomes. Data will be presented in tables as illustrated in Table 2 and supplemented with charts and graphs to enhance clarity and highlight patterns.

**Table 2:** Interventions to Mitigate/Prevent Depression Among Adolescents and Young Adults 10- 24years.

**Author(s)** **Year Country/** **Region**	**Study Design**	**Setting**	**Sample Size**	**Measurement scale**	**Age Range**	**Target Condition (Anxiety/** **Depression/** **Both**	**Type of Intervention**	**Intervention Characteristics**			**Key Findings/ Recommendations**
								**Mode of Delivery**	**Duration of Intervention**	**Intensity, adherence, fidelity**	

The analysis will map intervention characteristics, including modality, duration, delivery format, and outcome measures used to assess effectiveness. Where reported, barriers and facilitators to implementation and access will be synthesized. Recommendations identified in the literature will be categorized thematically, including policy implications and service delivery improvements.

The findings will likely reveal various intervention types, including psychological therapies (e.g., cognitive-behavioral therapy), school-based programs, digital and mobile health interventions, and community-based initiatives. The review is also expected to identify key differences in intervention delivery methods, settings (e.g., clinical vs. non-clinical), and target populations (e.g., general population, high-risk youth, gender-specific groups).

The review may uncover emerging trends, such as increasing use of technology-enabled interventions (e.g., Apps, teletherapy), peer-led or youth-driven models, and interventions adapted for culturally diverse populations. In addition, the results may highlight knowledge gaps, such as limited interventions for younger adolescents, underrepresentation of low- and middle-income country settings, intervention types, and methodological limitations, or limited evaluation of long-term outcomes and cost-effectiveness. Finally, the identified gaps will inform future research directions.

Ultimately, the results will provide valuable insights for researchers, practitioners, and policymakers by mapping the scope and characteristics of existing evidence, identifying where evidence is lacking, and informing future intervention design and research priorities.

## Discussion

This scoping review maps the literature on non-pharmacological interventions aimed at reducing anxiety and depression symptoms among adolescents and young adults (10–24 years), a developmental period often inconsistently defined in prior syntheses. By focusing specifically on this age range, the review aims to address fragmentation in the literature where adolescents and young adults are frequently grouped with children or older adults, limiting developmental specificity. It aims to highlight findings on interventions commonly adopted among this age group and identify well-established and emerging strategies by systematically examining the types of interventions, delivery contexts, and reported outcomes and implementation characteristics. It will also help highlight gaps in the literature including mobile-based tools, and school-based programs in low-resource settings. These findings will support future efforts to design, implement, and evaluate interventions that are developmentally appropriate, easily scalable and accessible.

Furthermore, the choice of a scoping review methodology will allow for the inclusion of diverse study designs and literature, capturing a wide range of evidence that might otherwise be excluded in more narrowly focused systematic reviews [22]. The findings of this review will be particularly relevant to policymakers, and educators who are interested in the mental wellbeing of the adolescents and young adults. Ultimately, this scoping review will contribute to advancing the well-being and knowledge in adolescents and young adult mental health by providing a structured overview of existing interventions, identifying gaps in the literature, and laying the foundation for future targeted reviews or primary research.

### Limitations

While this scoping review is designed to provide a comprehensive overview of interventions for anxiety and depression among adolescents and young adults, several limitations must be acknowledged. First, although the review will employ a broad and inclusive search strategy across multiple databases, it will be limited to studies published in English due to resource constraints. This may result in the exclusion of relevant interventions reported in other languages, particularly those implemented in non-English-speaking regions. Grey literature (e.g., reports, theses, policy briefs) will be excluded as this is often not peer-reviewed. Though grey literature may have resulted in some additional papers, we aim to synthesize evidence grounded in established academic standards, hence limiting inclusion to peer-reviewed studies helps ensure methodological transparency and credibility, thus excluding grey literature rather helps the scientific rigor.

Second, variations in the definitions and measurement of “adolescence” and “young adulthood” across studies may pose challenges in age-group categorization. Although efforts will be made to include studies where participants fall within the 10–24 age range, some degree of inconsistency may remain. Similarly, interventions that target general youth populations without disaggregated data may limit specific age-related insights.

Third, consistent with scoping review methodology, no formal critical appraisal of included studies was conducted; therefore, the quality and risk of bias of the evidence base were not systematically assessed. As a result, the findings should be interpreted with caution, as they reflect the breadth of available literature rather than the strength or reliability of the evidence.

Fourth, this review was restricted to studies published in the last 10 years to capture the most current evidence and practices. While this enhances relevance, it may have excluded older relevant studies due to publication bias, potentially overlooking foundational work. There may also be differences between included studies, such as differences in study design and instruments or measures of disease which may impact the prevalences of anxiety or depression reported and hence, the effectiveness of the intervention [24,30].

The review will aim to transparently address these limitations to ensure accurate interpretation of findings and to inform recommendations for future research.

## Conclusion

This scoping review will provide a comprehensive mapping of the current literature on interventions aimed at preventing or reducing symptoms of anxiety and depression among adolescents and young adults. Given the increasing global burden of mental health issues in this age group and the growing diversity of intervention types and delivery settings, this review is both timely and necessary. By identifying and synthesizing the existing evidence, the review will offer valuable insights into the range of available interventions, identify and highlight key areas of innovation, and critical gaps in the literature. These findings may serve as a foundational resource for researchers, policymakers, clinicians, educators, and mental health service planners, guiding future research, program development, and policy initiatives aimed at improving mental health outcomes for adolescents and young adults.

### Ethics and dissemination

Ethics approval is not required as this scoping review will include only already published literature and will not involve human participants. The results of this review will be disseminated through publication in a peer-reviewed journal, and presentation at relevant conferences.
